# Supplementary material for: A multiplexed bioluminescent reporter for sensitive and non-invasive tracking of DNA double strand break repair dynamics in vitro and in vivo
Source: Nucleic Acids Res. 2020 Aug 14;48(17):e100. doi: 10.1093/nar/gkaa669 (PMC7515717; doi:10.1093/nar/gkaa669)
Supplement: gkaa669_Supplemental_File [file gkaa669_supplemental_file.pdf]

Supplementary Information

A multiplexed bioluminescent reporter for sensitive and non-invasive tracking of DNA double strand break repair dynamics *in vitro* and *in vivo*

Jasper Che-Yung Chien[1], Elie Tabet[2, 3], Kelsey Pinkham[2], Cintia Carla da Hora[2,4], Jason Cheng-Yu Chang[5,6], Steven Lin[5,6], Christian Elias Badr[2,4*], Charles Pin-Kuang Lai[1,7,8*]

[1]Institute of Atomic and Molecular Sciences, Academia Sinica, Taipei 10617
[2]Department of Neurology, Massachusetts General Hospital, Boston MA
[3]Department of Biomedical Engineering, University of South Dakota, 4800 N. Career Ave, Suite 221, Sioux Falls, SD USA
[4]Neuroscience Program, Harvard Medical School, Boston MA
[5]Institute of Biochemical Sciences, National Taiwan University, Taipei, Taiwan
[6]Institute of Biological Chemistry, Academia Sinica, Taipei, Taiwan
[7]Chemical Biology and Molecular Biophysics Program, Taiwan International Graduate Program, Academia Sinica, Taipei, Taiwan
[8]Genome and Systems Biology Degree Program, National Taiwan University and Academia Sinica, Taipei, Taiwan

[*]C.E.B and C.P.L contributed equally to this work.

[*]To whom correspondence should be addressed:
Christian E. Badr, Tel: 1-617-643-3485; Fax: 1-617-724-1537; Email: badr.christian@mgh.harvard.edu
Charles P. Lai, Tel: 886-2-2366-8204; Fax: 886-2-2362-0200; Email: laicharles@sinica.edu.tw

Present Address:
Christian E. Badr, Department of Neurology, Massachusetts General Hospital, 149 13th Street, Charlestown, MA, 02129, USA.

Charles P. Lai, Institute of Atomic and Molecular Sciences, Academia Sinica, No. 1, Roosevelt Rd., Sec. 4, Taipei 10617, Taiwan.

Supporting Information

2 Supplementary Tables

8 Supplementary Figures

Supplementary Table 1. List of primers used in this study

Primer Name	5' to 3'
gRNA-1-fwd	CACCGCGACGATCGCGAGATGTTAT
gRNA-1 rev	AAACATAACATCTCGCGATCGTCGC
gRNA-2-fwd	CACCGTTATAGGTGTAGGGATAAC
gRNA-2-rev	AAACGTTATCCCTACACCTATAAC
gRNA-3-fwd	CACCGTAACAGGGTAATTCGATCTG
gRNA-3-rev	AAACCAGATCGAATTACCCTGTTAC
gRNA-4-fwd	CACCGTTATAGGTGTAGGGATAACA
gRNA-4-rev	AAA CTGTTATCCCTACACCTATAA C
gRNA-5-fwd	CACCGCGCGAGATGTTATAGGTGTA
gRNA-5-rev	AAA CTACACCTATAACATCTCGCGC
trG-fwd	TTTAGTGAACCGTCAGATCCAAGCTGGCTGCAC
trG-rev	ATTCGAAGCTTGAGCTCGAGCTAGTC
gRNA-6-fwd	CACCGTCGCGAGATGTTATAGGTGT
gRNA-6-rev	AAA CACACCTATAACATCTCGCGAC
NGS-fwd	TCGTCGGCAGCGTCAGATGTGTATAAGAGACAGGCTC AAAGAGATGGAAGCCA
NGS-rev	GTCTCGTGGGCTCGGAGATGTGTATAAGAGACAGCCC TTGATCTTGTCCACCTGG
TIDE-1-fwd	GAAGACTTCAACATCGTGGC
TIDE-2-Rev	TATTTTCGCAAAGTCCGTCG
TIDE-2-fwd	GACACCGACTCTAGTCCA
TIDE-2-rev	TCA CCG GAA TAA GCT AGT CG

Supplementary Table 2. ***In silico*** **scores for different gRNAs**

gRNA number	Benchling On-Target Score	CHOPCHOP Efficiency
1	40.66	40.67
2	24.44	24.44
3	65.3	65.3
4	57.03	57.04
5	54.05	54.05
6	49.2	49.24

The insertion sequence in BLRR was analysed by Benchling (https://benchling.com) and CHOPCHOP (https://chopchop.cbu.uib.no/).

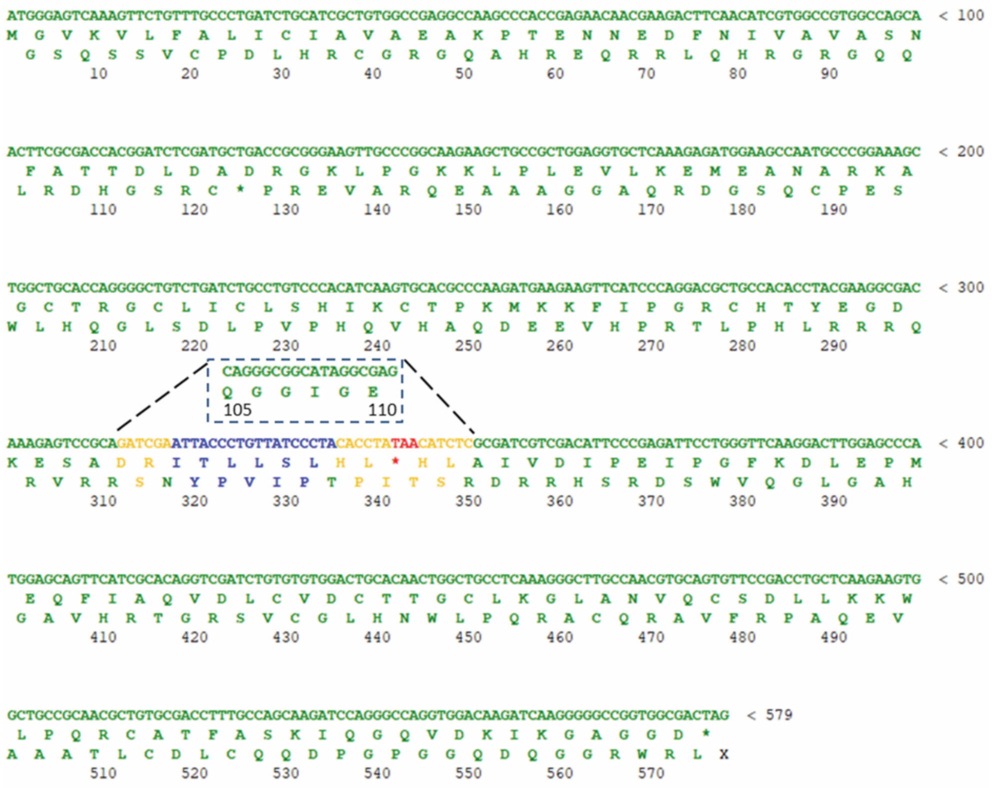

Supplementary Figure 1. Sequence map of the silence-mutated Gluc sequence in BLRR. Green, Gluc; yellow, spacer; red, stop codon; blue, I-SceI target site; dashed line, wild-type Gluc. Gluc, *Gaussia* luciferase

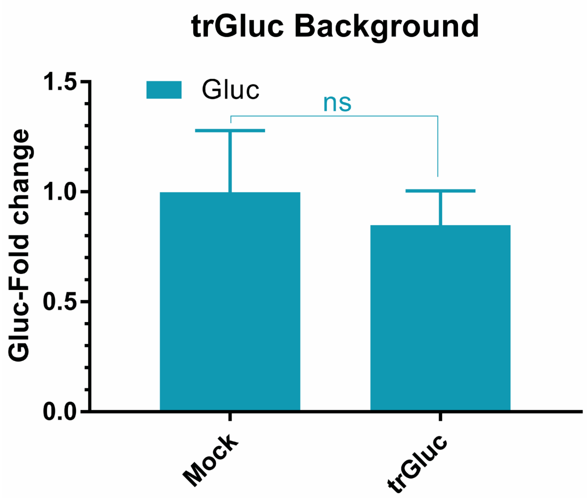

Supplementary Figure 2. trGluc does not exhibit Gluc activity. BLRR cells transfected with trGluc do not exhibit Gluc activity compared with mock controls. The Gluc signal was normalised against the mean value for mock controls. Data are presented as mean ± SEM of three biological replicates (p >0.05; two-tailed Student t-test; ns, non-significant). Gluc, *Gaussia* luciferase; trGluc, truncated Gluc.

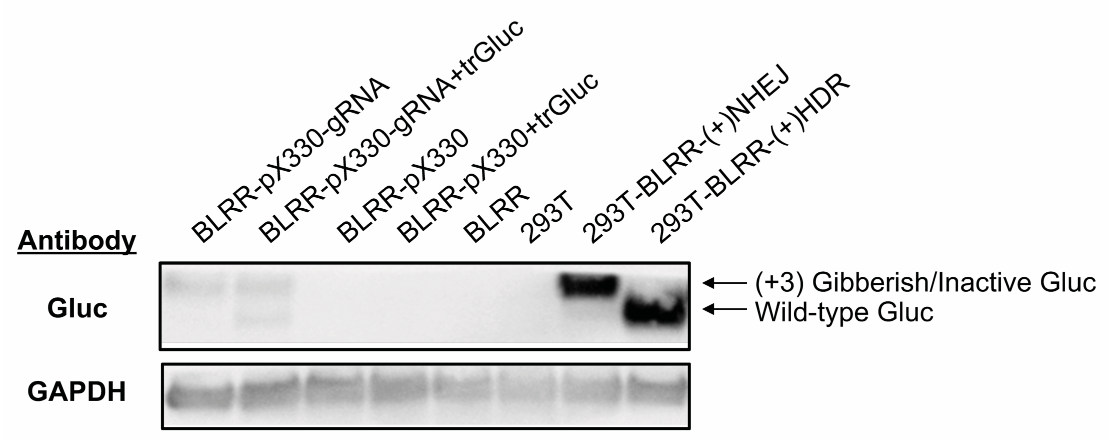

Supplementary Figure 3. Western blotting analysis showing Gluc and T2A peptide expression following HDR and NHEJ repair events in BLRR cells. Wild-type Gluc, 19.86 kDa; (+3) Gibberish, 23.17 kDa. BLRR cells were transfected with pX330-gRNA with or without trGluc. 293T cells transfected with BLRR-(+)NHEJ or BLRR-(+)HDR served as controls for NHEJ (*i.e.* (+3) gibberish Gluc and HDR (*i.e.* wild-type Gluc), respectively. BLRR-pX330, BLRR-pX330+trGluc, BLRR and wild-type 293T (293T) served as negative controls. GAPDH was immunoprobed as a loading control. BLRR, bioluminescent; trGluc, truncated Gluc.

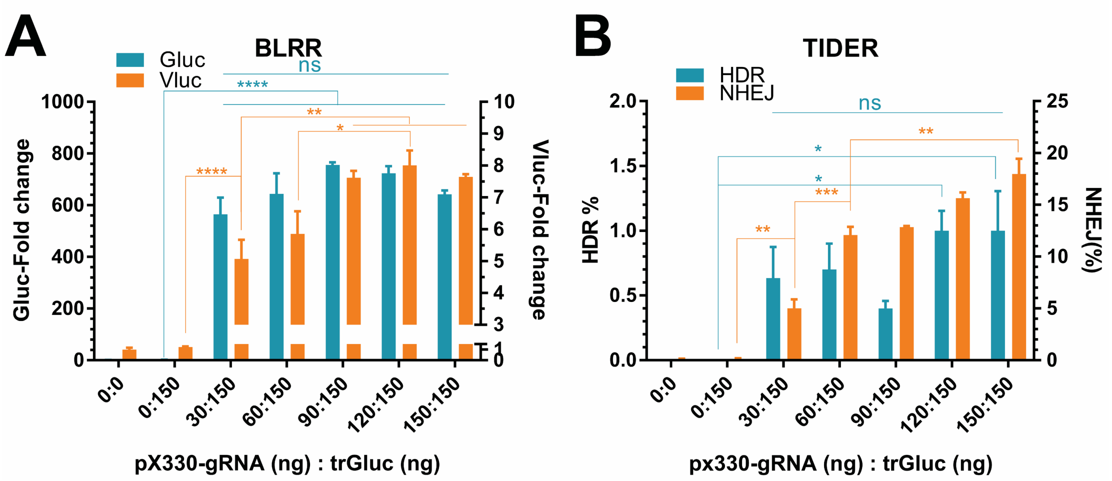

Supplementary Figure 4. BLRR and TIDER analyses of BLRR cells transfected with increasing amounts of pX330-gRNA reveals an increase in NHEJ events. (**A**) BLRR analysis reveals a pX330-gRNA dose-dependent increase in Vluc while elevated Gluc remains unchanged. BLRR cells were transfected with a fixed amount of trGluc and increasing quantities of pX330-gRNA. (**B**) TIDER analysis of the BLRR cells from (A) reveals a dose-dependent increase in NHEJ events. Data are presented as mean ± SEM of three biological replicates. Significance was calculated by one-way ANOVA followed by Tukey's post-hoc test (**p* <0.05, ***p* <0.01, ****p* <0.001, **** *p* <0.0001).

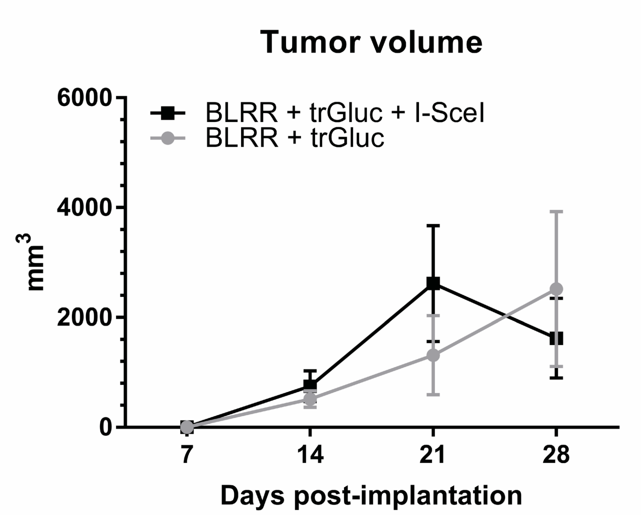

Supplementary Figure 5. Sizes of implanted tumours over time. Tumour growth was monitored over time by calliper measurement. Data are presented as mean ± SEM of three mice. BLRR, bioluminescent repair reporter; trGluc, truncated Gluc.

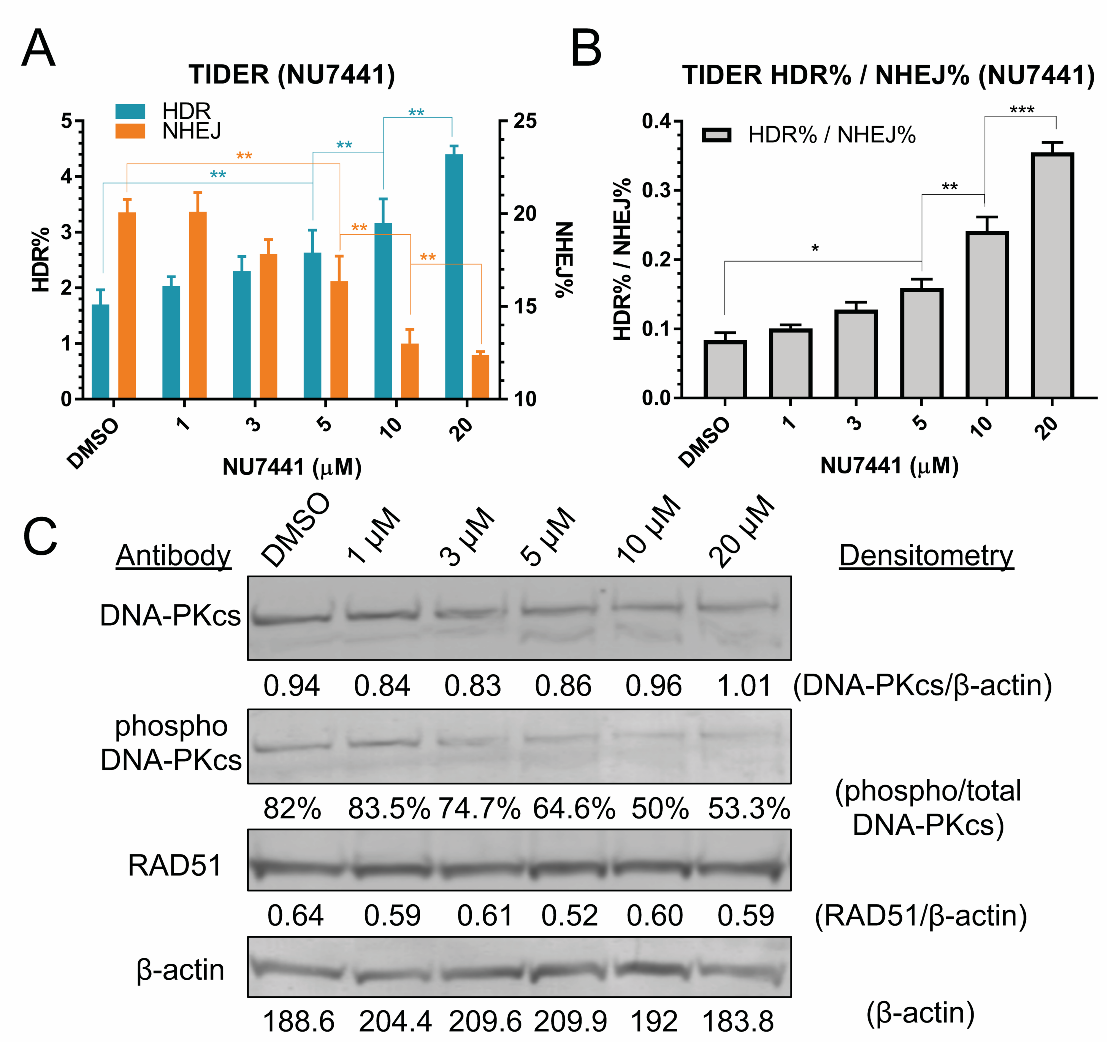

Supplementary Figure 6. TIDER and western analysis of the HDR-enhancing effect. (**A**) NU7441-treated BLRR cells showing a dose-dependent increase in HDR events and a decrease in NHEJ events. TIDER analysis was performed on the same cells used to generate the results shown in Figure 6A. (**B**) HDR% and NHEJ% values reveal a dose-dependent increase in HDR events. Data are presented as mean ± SEM of three biological replicates. **(C)** Western blot analysis reveals a dose-dependent decrease in the abundance of phosphorylated DNA-PKcs. Significance was calculated by one-way ANOVA compared as indicated, followed by Tukey's post-hoc test (**p* <0.05, ***p* <0.01, ****p* <0.001, *****p* <0.0001). HDR, homology-directed repair; NHEJ, non-homologous end joining; TIDER, tracking of insertions, deletions and recombination events; DNA-PKcs, DNA-dependent protein kinase catalytic subunit.

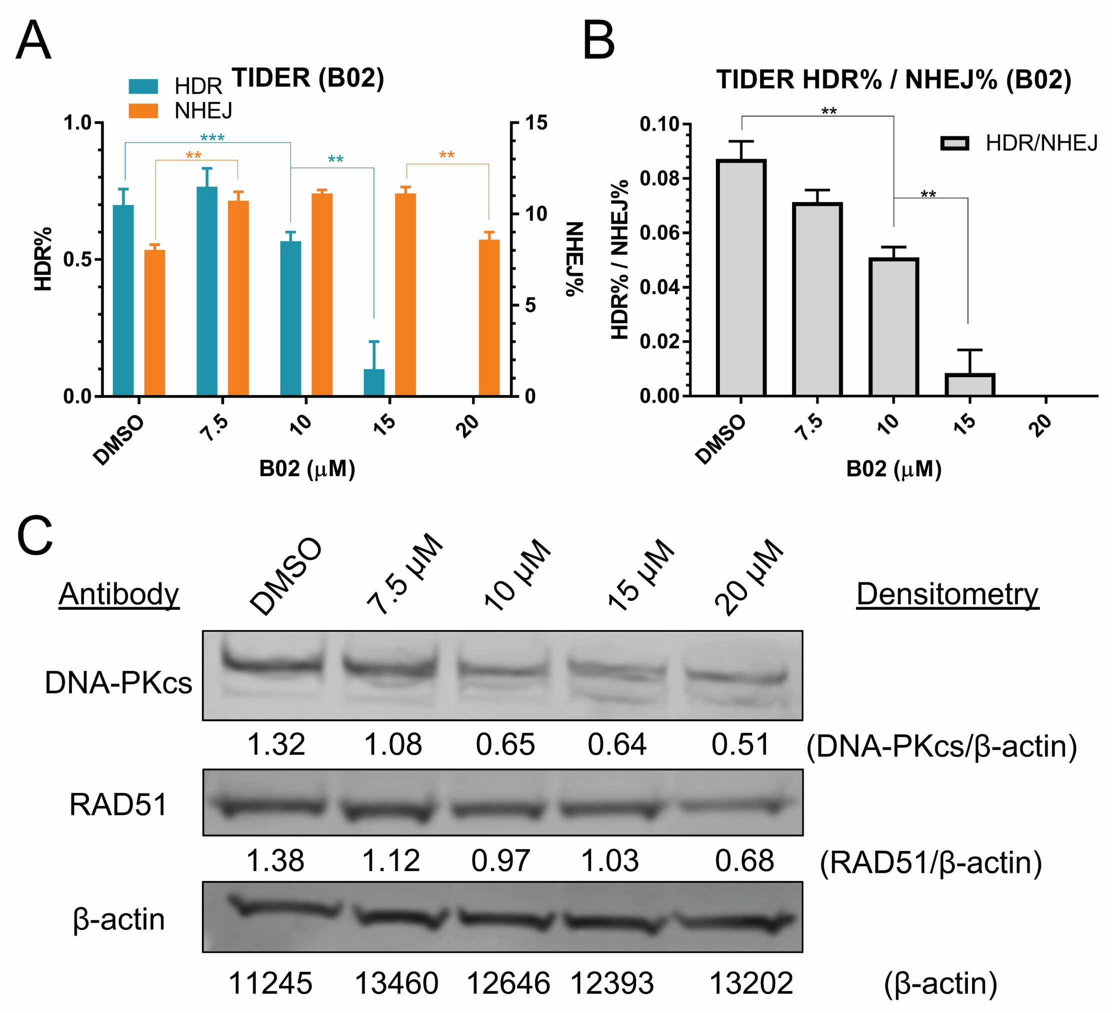

Supplementary Figure 7. TIDER and western analysis of the HDR-inhibiting effect. (**A**) B02-treated BLRR cells showing a dose-dependent decrease in HDR events. TIDER analysis was performed on the same cells used to generate the results shown in Figure 6D. (**B**) HDR% and NHEJ% values reveal a dose-dependent decrease in HDR events. Data are presented as mean ± SEM of three biological replicates. **(C)** Western blot analysis reveals a dose-dependent decrease in the abundance of RAD51 and DNA-PKcs. Significance was calculated using one-way ANOVA compared as indicated, followed by Tukey's post-hoc test (**p* <0.05, ***p* <0.01, ****p* <0.001, *****p* <0.0001). HDR, homology-directed repair; NHEJ, non-homologous end joining; TIDER, tracking of insertions, deletions and recombination events; DNA-PKcs, DNA-dependent protein kinase catalytic subunit.

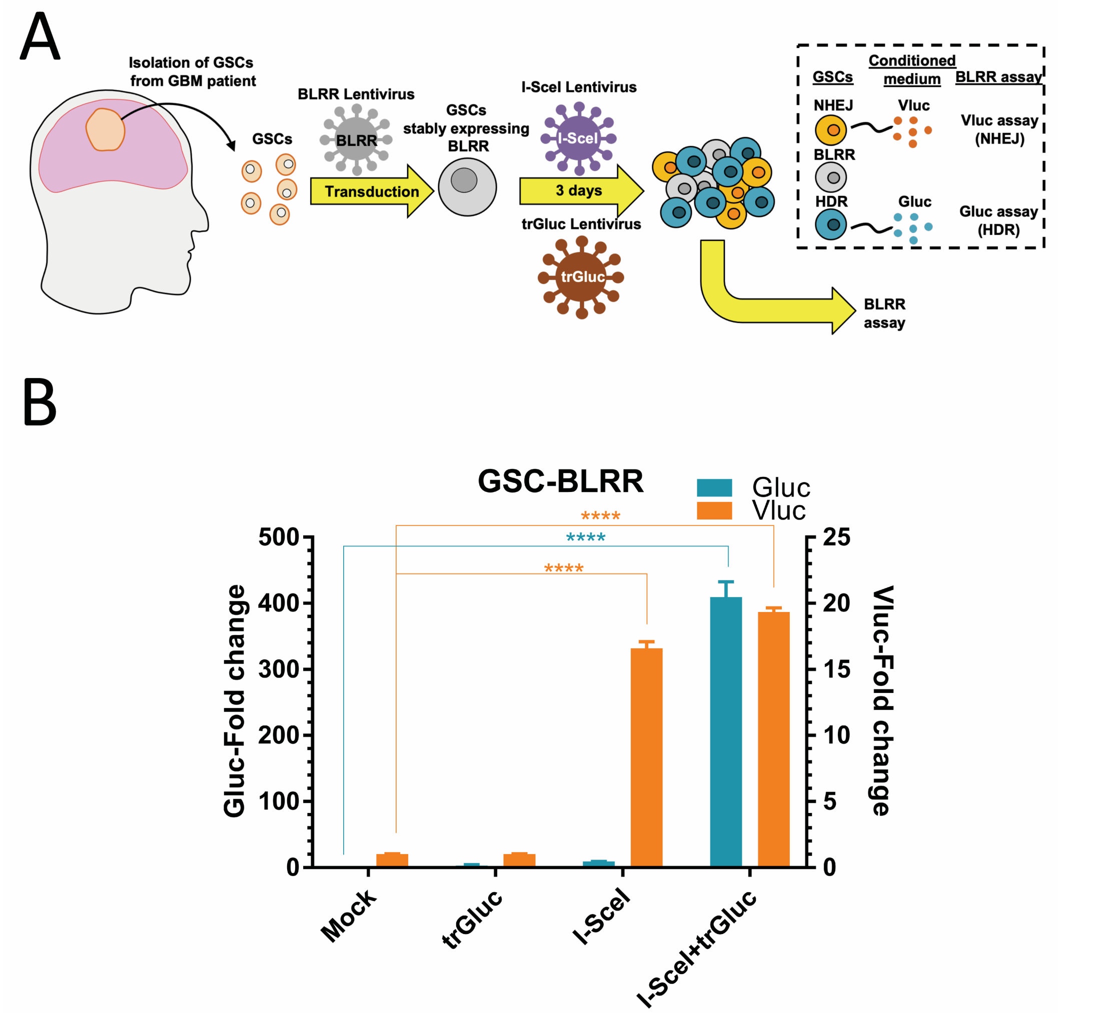

Supplementary Figure 8. BLRR reporter controls in patient-derived GSCs. (**A**) Schematic diagram of the BLRR assay in patient-derived GSCs. GSCs from patients were stably transfected with BLRR, then sequentially transfected with lentiviruses encoding trGluc and I-SceI, then assessed by BLRR assay. (**B**) BLRR assays demonstrate DSB repair induced by I-SceI in patient-derived GSCs. I-SceI+trGluc yields a significant increase in Gluc and Vluc signals, whereas I-SceI only yields an increase in Vluc activity, revealing BLRR specificity for reporting HDR and NHEJ events in GSCs. Mock and trGluc groups served as negative controls. Data were normalised against mock controls and results are presented as mean ± SEM of three biological replicates. Significance was calculated using one-way ANOVA compared to mock controls, followed by Tukey's post-hoc test (*****p* <0.0001). BLRR, bioluminescent repair reporter; GBM, glioblastomas; Gluc, *Gaussia* luciferase; GSCs, GBM cancer stem cells; HDR, homology-directed repair; trGluc, truncated Gluc; Vluc, *Vargula* luciferase.
